# Is the replication crisis a base-rate fallacy?

**DOI:** 10.1007/s11017-022-09561-8

**Published:** 2022-02-27

**Authors:** Bengt Autzen

**Affiliations:** Department of Philosophy, University College Cork, Cork, Ireland

**Keywords:** Philosophy of medicine, Philosophy of science, Replication crisis, Base-rate fallacy, Bayesianism

## Abstract

Is science in the midst of a crisis of replicability and false discoveries? In a recent article, Alexander Bird offers an explanation for the apparent lack of replicability in the biomedical sciences. Bird argues that the surprise at the failure to replicate biomedical research is a result of the fallacy of neglecting the base rate. The base-rate fallacy arises in situations in which one ignores the base rate—or prior probability—of an event when assessing the probability of this event in the light of some observed evidence. By extension, the replication crisis would result from ignoring the low prior probability of biomedical hypotheses. In this paper, my response to Bird’s claim is twofold. First, I show that the argument according to which the replication crisis is due to the low prior of biomedical hypotheses is incomplete. Second, I claim that a simple base-rate fallacy model does not account for some important methodological insights that have emerged in discussions of the replication crisis.

## Introduction

The replication crisis refers to the subject of a methodological debate issuing from the observation that the outcomes of a number of scientific studies are difficult or impossible to replicate on subsequent investigation. The biomedical sciences are doubly affected by the replication crisis. On the one hand, medical research figures prominently in the discussion about the lack of replicability of scientific studies. Indeed, it has been argued that most published medical research is false [1]. On the other hand, physicians and patients rely on the accuracy of the published results in medicine in order to make treatment decisions on their basis. As a result, concerns regarding the reliability of medical research can have a direct impact on drug development and public health policy.

To illustrate, consider a study by Glenn Begley and Lee Ellis, in which scientists at the biotechnology firm Amgen tried to replicate 53 high-impact findings in pre-clinical cancer research but could confirm the findings of only 6 studies (11%) [2]. These results appear to be consistent with those of others in the pharmaceutical industry. For instance, a team at Bayer HealthCare reported that only about 25% of published preclinical studies could be replicated [3]. Furthermore, Begley and Ellis lament that some non-replicable findings have given rise to a large secondary literature that expands on aspects of the original observation without seeking to confirm or falsify its fundamental basis.

In a recent paper, Alexander Bird offers an explanation for the apparent lack of replicability in the biomedical sciences [4]. Bird argues that the surprise—or some might say, shock—at the failure to replicate biomedical research is a result of the fallacy of neglecting the base rate. The base-rate fallacy arises in situations in which one ignores the base rate—or prior probability—of an event when assessing the probability of this event in the light of some observed evidence. By extension, according to Bird, the replication crisis results from ignoring the low prior probability of biomedical hypotheses.

In this paper, my response to Bird is twofold. First, I show that Bird’s argument that the replication crisis is due to the low prior of biomedical hypotheses is incomplete. In order to make the case that the observed lack of replicability in the biomedical sciences is due to the low prior probability of biomedical hypotheses, a more fine-grained analysis of these prior probabilities in combination with an empirical rationale for their assignment is needed. Second, I argue that it is questionable that a simple base-rate fallacy model could sufficiently account for the replication crisis, since it ignores some crucial methodological insights, such as the role of selective reporting, that have emerged in discussions about the replicability of biomedical studies.

## Base-rate fallacy model

The base-rate fallacy is typically discussed in the context of medical diagnosis. Suppose a patient begins displaying symptoms of an extremely rare, terminal disease, present in only 0.1% of the population. The patient, seeking a diagnosis and treatment, goes to the doctor to have a test done. The doctor runs a highly accurate test and it comes out positive. How probable is it that the patient has the disease?

In order to address this question, some notation has to be introduced. Let *H* denote the hypothesis that the patient has the disease and *T* denote the event that the test comes out positive. The prior probability of the patient’s having the rare disease is assumed to be 0.001 (i.e., *P*(*H*) = 0.001), since 0.1% of the population carries the disease. This assignment of prior probabilities is typically seen as uncontroversial insofar as there are empirical frequency data about the prevalence of disease in a population on hand. The probability of the test coming out positive given that the patient has the disease is assumed to be equal to 0.97 (i.e., *P*(*T*|*H*) = 0.97). Further, the probability of the test coming out negative given that the patient does not have the disease is assumed to be equal to 0.9 (i.e., *P*(¬*T*|¬*H*) = 0.9). These numerical values imply that the probability of the test coming out positive given that the patient does not have the disease equals 0.1 (i.e., *P*(*T* |¬*H*) = 0.1). This probability is referred to as the ‘false positive’ rate of the test. Similarly, the probability of the test coming out negative given that the patient does have the disease equals 0.03 (i.e., *P*(¬*T*|*H*) = 0.03). This probability is referred to as the ‘false negative’ rate of the test. The probability of the test coming out positive, *P*(*T*), is given by *P*(*T*|*H*)*P*(*H*) + *P*(*T*|¬*H*)*P*(¬*H*). Based on these assumptions, per Bayes’s theorem, the probability that the patient has the disease given that the test has come out positive is as follows:

$$P(H|T)~ = ~\frac{{P(T|H)P(H)}}{{P(T)}}~ \approx ~0.01$$

The upshot is that even though the test has fairly low false positive and false negative rates, the probability of having the disease given a positive test result is very low. This result is due to the low prior probability of having the disease. Making inferences regarding the probability of having the disease based alone on the error rates of the test while neglecting the prior probability (or base rate) of having the disease amounts to a flawed form of probabilistic reasoning.

In order to apply this mathematical model to the replication crisis, some terminology on null hypothesis significance testing also needs to be introduced. Suppose scientists would like to investigate a particular research hypothesis, such as exercise leads to weight loss. In a significance test, one does not directly address this hypothesis of interest but rather evaluates the ‘null hypothesis’ that exercise is unrelated to weight loss. One calculates the *p*-value, that is, the probability of observing a sample realisation that would have given rise to a value of the test statistic greater than or equal to the one actually observed under the assumption that the null hypothesis is true. In a significance test, the false positive rate (i.e., the probability of rejecting a true null hypothesis) is fixed at some small number, usually 0.05 or 0.01, which is called the ‘significance level’ of the test and is denoted by *α*. If the *p*-value is smaller than *α*, then the null hypothesis is rejected. Otherwise the null hypothesis is not rejected. The false negative rate, that is, the probability of accepting a false null hypothesis, is denoted by *β*. The ‘power’ of a test refers to the term 1 − *β*. Tests with a power of 0.8 or higher are typically considered to be adequately powered in biomedical research.^1^

With this terminology in mind, I now turn to Bird’s diagnosis of the replication crisis. By close analogy to the base-rate fallacy model, Bird argues that ‘the surprise at the number of failed replications and consequent sense of crisis are a result of the fallacy of neglecting the base rate’ [4, p. 965]. More specifically, he claims that biomedical hypotheses have a low prior probability of being true just as the hypothesis that the patient has the rare disease has a low prior probability of being true in the base-rate fallacy example above. Due to the effect of this low base rate, published significant results in the biomedical literature also have a low probability of being true. For instance, if only 1% of tested hypotheses are true and a false positive rate of 0.05 and false negative rate of 0.2 are assumed (i.e., *α* = 0.05, *β* = 0.2), then 86% of published significant results will be false positives. The reason for this high rate of published false positives is that 5% of a very large number of false hypotheses is still larger than 80% of a very small number of true hypotheses. If many more false hypotheses than true hypotheses are tested, the relative frequency of significant results associated with true hypotheses will still be low. The base-rate fallacy model then seems to offer a simple mathematical explanation for why one should expect a large number of false positives in the biomedical literature.

## Prior(s) of biomedical hypotheses

In order to make the case that biomedical hypotheses have low prior probabilities, Bird distinguishes between hypotheses that are derived from some more fundamental scientific theory and hypotheses that stem from sources other than theory. With regard to the former, Bird contrasts biomedical hypotheses with hypotheses in physics. He argues that our knowledge of physical systems is more complete than our knowledge of biological systems and that theories in physics enjoy stronger experimental support than theories in biomedicine. As a result, biomedical hypotheses derived from theory have a lower prior probability than hypotheses derived from physical theories. With regard to the latter, biomedical hypotheses stemming from sources other than theory may be drawn from the results of observational studies or, more problematically, by unsystematic observations or the researcher’s intuition. Bird suggests that these kind of hypotheses again have only a low prior probability.

I argue that Bird’s analysis of the prior probabilities of biomedical hypotheses is too coarse-grained. In particular, Bird does not explore the possibility that even when they are not grounded in an established scientific theory, biomedical hypotheses can have significantly different prior probabilities due to their differing empirical support. Take, for instance, one of the first modern meta-analyses of randomised controlled trials, which addresses the use of antibiotic prophylaxis compared to no treatment in colon surgery. In it, Mark Baum et al. analyse 26 trials published from 1965 to 1980 and find that there is strong evidence that antibiotic prophylaxis reduces postoperative morbidity from colon surgery [8]. However, as argued by John Ioannidis and Joseph Lau, the efficacy of antibiotic prophylaxis could have been identified as early as 1971 after the publication of the first five studies, which taken together involved about 300 patients [9]. In this way, Ioannidis and Lau’s assessment suggests that by the time Baum et al. published their meta-analysis in 1981, there already existed strong evidential support in favour of the hypothesis that antibiotic prophylaxis is efficacious, thereby warranting the assignment of a high prior probability to this hypothesis.

Such strong evidential support for a biomedical research hypothesis, however, cannot generally be assumed. For instance, hypotheses that are generated by exploratory analyses in risk-factor epidemiology are frequently published with the idea that they can be subjected to further causal assessment [10]. In the absence of further evidence from toxicology, animal studies, or other disciplines that could potentially offer support, a high prior probability seems to be unwarranted for such hypotheses generated by ‘black-box’ epidemiology [11, 12].

Identifying an adequate reference class for a hypothesis is essential when making inferences based on the base-rate fallacy model. Returning to the original context of medical diagnosis, it can be misleading to assume base rates that are averaged across the whole population. To illustrate, consider a sexually active middle-aged gay man living in London who is being tested for HIV. Assume that the test has the error rates assumed in the previous example—that is, a false positive rate of 0.1 and a false negative rate of 0.03. What is the prior probability of having HIV? Phrased differently, what is an adequate reference class for calculating the relative frequency of HIV?

One could adopt the prevalence of HIV among all men in the United Kingdom: 2.3 per 1000 [13, p. 8]. Alternatively, one could use the prevalence of HIV among gay/bisexual men in London: 135 per 1000 [13, p. 8]. Given the error rates of the test, the post-test probability of having HIV will depend crucially on the choice of reference class. If the larger reference class is chosen and, hence, a prior probability of having HIV of 0.0023 is estimated, the post-test probability of having the virus will be 0.02; if the more specific reference class is chosen and, hence, a prior of 0.135 is estimated, the post-test probability of having HIV will be 0.6. In other words, the post-test probability of having HIV is approximately 30 times larger when the more specific reference class is chosen to estimate the base rate of having HIV. In this example, it seems misleading to use the whole British male population when calculating the prior probability of the patient’s having HIV. Doing so would ignore available HIV prevalence data that identify gay/bisexual men in London as a sub-population with a higher risk of being infected with HIV.

Returning to the replication crisis in biomedical research, it seems misleading to talk about the prior probability of biomedical hypotheses in general. Rather, an adequate Bayesian analysis of the replication crisis should allow for different kinds of biomedical hypotheses to have different chances of being true. An analysis of that kind has been done by Ioannidis [1]. Ioannidis broadly distinguishes between hypotheses assessed in randomised controlled trials, meta-analyses, exploratory epidemiological studies, and discovery-oriented exploratory research with massive testing. He posits dramatically different prior probabilities for hypotheses in these distinct research designs—for example, assigning discovery-oriented exploratory research with massive testing a prior of $\frac{1}{{1001}}$ but assigning meta-analyses priors ranging between $\frac{1}{{4}}$ and $\frac{2}{{3}}$ (see Table 1). Ioannidis’s priors cannot be considered empirically based in the sense that they result from relative frequencies in real data sets. However, these priors can still be called empirically motivated insofar as they aim to capture the idea that different classes of biomedical hypotheses warrant different prior degrees of belief. Indeed, even some critics of Ioannidis’s argument, such as Steven Goodman [14] seem to be sympathetic to Ioannidis’s empirically motivated prior probabilities of biomedical hypotheses.

**Table 1:** Prior probabilities of biomedical hypotheses

Research design	Prior
Randomised controlled trial	$\frac{1}{{6}}$–$\frac{1}{{2}}$
Meta-analysis	$\frac{1}{{4}}$–$\frac{2}{{3}}$
Exploratory epidemiological studies	$\frac{1}{{11}}$
Discovery-oriented exploratory research	$\frac{1}{{1001}}$

In response to Ioannidis’s classification, one might wonder how the prior probability of a hypothesis can change depending on the method by which it is examined. The guiding idea here seems to be that in order to be made the subject of, say, a meta-analysis, a hypothesis has already been examined by a number of previously published studies. In that case, the prior of a hypothesis is really just an informal posterior. That is, rather than explicitly calculating a posterior in a Bayesian analysis based on the available studies, one estimates the prior probability of the hypothesis in the light of previous results in a non-formal way. In addition, a number of previous studies typically support the hypothesis under consideration; otherwise there would be little motivation to pursue a meta-analysis on the topic. Although there is no reason to think that the method of investigating a hypothesis can *in itself* change the prior probability of a hypothesis, empirical regularities suggest that hypotheses assessed by means of a meta-analysis typically enjoy stronger prior support than, say, a hypothesis subjected to an exploratory epidemiological study.

Suppose one adopts the prior probabilities in Table 1. What are the implications for the base-rate fallacy model? Not too surprisingly, the post-test probability that a biomedical hypothesis is true will depend on the class of hypothesis. For instance, while an adequately powered RCT with 0.5 prior probability has a post-test probability value (PPV) of 0.94, an adequately powered exploratory epidemiological study with a prior probability of 0.09 has a PPV of only 0.62 (see Table 2). In the model, an adequately powered meta-analysis with a prior probability of 0.25 comes out with a PPV of 0.84. At the other end of the spectrum, one finds the post-test probability of a hypothesis generated by discovery-oriented exploratory research with a negligible PPV of 0.004 given a prior probability of $\frac{1}{{1001}}$. Note, however, that in the case of discovery-oriented research, it is assumed that the test procedure is underpowered (i.e., *β* = 0.8). So the picture that results from this analysis is quite diverse. In any case, not every type of biomedical hypothesis will have a low post-test (or post-publication) probability of being true. To the contrary, the claim that most published research findings in the biomedical literature are false cannot be substantiated by means of the base-rate fallacy model in combination with Ioannidis’s priors, if one focuses exclusively on randomised controlled trials, meta-analyses, and exploratory epidemiological studies.

**Table 2:** Assumptions and results of the base-rate fallacy model

Research design	Prior	*α*	*β*	PPV
Randomised controlled trial	0.5	0.05	0.2	0.94
Meta-analysis	0.25	0.05	0.2	0.84
Exploratory epidemiological studies	$\frac{1}{{11}}$	0.05	0.2	0.62
Discovery-oriented exploratory research	$\frac{1}{{1001}}$	0.05	0.8	0.004

While the present analysis suggests taking a more fine-grained view on the prior probabilities of biomedical hypotheses when applying the base-rate fallacy model, one might object that Bird is, effectively, talking about an *average* prior probability of biomedical hypotheses. Based on this view, the use of a single prior for biomedical hypotheses is unproblematic. However, in order to make the case that the prior of biomedical hypotheses—understood as the average prior probability of biomedical hypotheses—is responsible for the replication crisis, a further explanatory step is needed. Specifically, doing so requires demonstrating that the weighted average prior probability of biomedical hypotheses is sufficiently low to result in an observed low post-test probability in the base-rate fallacy model. That is, one would have to take into account the proportion of the different types of hypotheses in the biomedical literature or, maybe more specifically, some sub-field within biomedicine (e.g., pre-clinical cancer research) or some particular biomedical journal. These proportions can then be used to assign different weights when averaging over the priors of the different types of biomedical hypotheses. So the average prior of a biomedical hypothesis would depend not only on the priors assigned to the individual hypothesis classes but also on the proportion of these hypotheses discussed in the biomedical literature. It remains an open question as to whether this additional analysis, taking into account both the prior probability of different kinds of biomedical hypotheses as well as their representation in the biomedical literature, can support Bird’s claim regarding the role of the prior of biomedical hypotheses in the replication crisis. In any case, the moral remains that one cannot explain a quantitative phenomenon, such as the rate of failed replications in the biomedical sciences, by means of a Bayesian model without detailed quantitative assumptions.

## Bias

Having argued that Bird’s account does not, as it stands, provide a satisfactory explanation for the replication crisis, one might ask whether the simple base-rate fallacy model needs to be enriched by other parameters to account for the current replication rates in the biomedical sciences. A candidate for such an additional model parameter is bias. In the context of the replication crisis, bias is generally understood as any systematic error that can over- or underestimate an intervention effect (e.g., [15]). Bird is sceptical of explanations of the replication crisis that make reference to bias; or, at least, he thinks that it is unnecessary to invoke bias since the low prior probability of biomedical hypotheses—or, more precisely, the combination of low prior of biomedical hypotheses and low significance level—is sufficient to explain the observed lack of replicability. Given the limitations of Bird’s explanation, however, a closer look at the potential role of bias in the replication crisis is indicated.

An early attempt to capture the influence of bias in a Bayesian model of the replication crisis is provided by Ioannidis [1].^2^ Introducing a bias parameter into a Bayesian model of the replication crisis helps to account for the claim that the greater a researcher’s degree of freedom—that is, the greater the flexibility in designs, definitions, and outcomes in a scientific field—the lower the probability that a published finding is true. Furthermore, Ioannidis shows that the probability that a published finding is true decreases when the proportion of reported significant results that are the artefact of some bias increases, unless the power of a test is less than or equal to its significance level.

In Bird’s explanation for the replication crisis, the prior probability of biomedical hypotheses plays a central role. Debates concerning the numerical value of the prior probability of a research hypothesis are notoriously difficult to resolve. There is, however, good reason to believe that bias plays an important role in accounting for the rate of published false positives in the biomedical sciences. Consider, for instance, the case of selective reporting (or ‘*p*-hacking’), understood as the conducting of alternative analyses on the same data set and then selectively reporting those that provide statistically significant support for a publishable claim. Eric Wagenmakers et al. consider scientists’ ability to fine-tune their analyses to the data to be the main factor for the replication crisis in the empirical sciences [18]. In line with this view, methodological research has accumulated evidence on the proportion of studies in which at least one primary outcome was changed, introduced, or omitted during the course of the analysis [19–22].

Providing an adequate account of the replication crisis in the biomedical sciences is not only of interest from a theoretical perspective but also has important implications for methodological reform in the empirical sciences. If selective reporting contributes significantly to the number of failed replications in biomedicine, then targeted interventions to improve statistical methodology in this field are needed. In contrast, generic calls for more basic research in the biomedical sciences in order to increase the prior probability of published research hypotheses, such as that by Bird [4], do not address this specific methodological issue.

Preregistration amounts to defining the research question and analysis plan before observing the research outcome. A number of initiatives have been put forward to incorporate preregistration into the publication process. For instance, the preregistration challenge provides researchers with a financial incentive to preregister their hypotheses before conducting the analysis of their study [23]. Preregistered studies avoid the problems associated with selective reporting, outcome switching, and other potential difficulties that result from blurring the line between exploratory and confirmatory research. In particular, preregistration makes sure that the methodological principle is warranted that in a hypothesis test, the data may be used only once. When researchers look for interesting patterns in a data set, they already use the data to help formulate a particular research hypothesis. The same data should then not be used to test the hypothesis that they helped to formulate [18]. In addition to being compelling based on general methodological considerations, there exists empirical support that preregistration dramatically reduces the rate of reported positive findings. Robert Kaplan and Veronica Irvin analyse randomised controlled trials between 1970 and 2012 funded by the National Heart, Lung, and Blood Institute on interventions aimed at decreasing the risk of cardiovascular disease and death [24]. In particular, they compare 30 studies published pre-2000 and 25 studies published post-2000. The year 2000 is of importance since the studies after this date were preregistered while the studies before this date were not. Kaplan and Irvine report that while 57% of the interventions studied pre-2000 show a positive effect, this number drops to 8% for the preregistered interventions investigated after 2000.

While the motivation for incorporating preregistration into the publication process becomes obvious when the role of bias, particularly selective reporting, is accounted for, it is less clear how such a policy can be motivated based on a simple base-rate fallacy model that ignores bias. Providing a comprehensive specification of the data analysis plan prior to observing the research outcome should not affect the prior probability of the research hypothesis under consideration. As a result, it is unclear why policy initiatives aimed at addressing selective reporting, such as preregistration, should play a central role in an ongoing methodological reform tackling the replication crisis if the main cause of the crisis is to be found in the low prior probabilities of biomedical hypotheses. The simple base-rate fallacy model therefore fails to account for some important methodological insights that have emerged in discussions of the replication crisis.

## Conclusion

Bird uses a simple base-rate fallacy model to emphasise the role that prior probabilities of biomedical hypotheses play in the replication crisis [4]. I have taken issue with two aspects of this account. First, I argued that Bird’s assignment of prior probabilities is too coarse-grained. Explaining a quantitative phenomenon such as the rate of false positives in the medical literature by means of a Bayesian model requires quantitative assumptions about the numerical prior probabilities of biomedical hypotheses. Without a justification for these prior probabilities, the attempted explanation of the replication crisis remains unsatisfactory. Second, I argued that a simple base-rate fallacy model does not do justice to some important methodological developments in response to the replication crisis. Selective reporting has become a main cause of concern in discussions of statistical methodology in the biomedical sciences. Preregistration—that is, the requirement adopted by a number of scientific journals to specify the research question and analysis plan prior to observing the research outcome—remains unmotivated if the diagnosis of the replication crisis focuses primarily on the role of the prior probabilities of biomedical hypotheses. I take this to be a deficit of a Bayesian model of the replication crisis that ignores bias.

## Footnotes

1 It has been argued that the current practice of null hypothesis significance testing is a hybrid form of statistical inference combining ideas from Ronald Fisher [5] and Jerzy Neyman and Egon Pearson [6]. For further discussion, see Gerd Gigerenzer [7].

2 Other mathematical models of the replication crisis that include bias can be found in Pfeiffer et al. [16] and Jager and Leek [17].
